# Myeloperoxidase and its negative relationship with cholesterol efflux capacity in patients with psoriasis: results from an observational cohort study

**DOI:** 10.1186/s12967-023-04506-8

**Published:** 2023-10-20

**Authors:** Elizabeth M. Florida, Haiou Li, J. Rodante, H. L. Teague, Martin P. Playford

**Affiliations:** Section of Inflammation and Cardiometabolic Diseases, National Heart, Lung and Blood Institute, National Institutes of Health, Bethesda, MD USA

## Dear Editor

Inflammation and oxidative stress can lower the functionality of apolipoprotein A-1 (ApoA1) and high-density lipoprotein (HDL) molecules, leading to defective reverse cholesterol transport and accelerating early atherosclerosis [1].

Myeloperoxidase (MPO) is an enzyme largely found in neutrophils, monocytes, and some macrophages that produces nitrating and chlorinating oxidants from hydrogen peroxide. While the positive impact of MPO production is the killing of unwanted microorganisms, studies have shown that increased levels of MPO can lead to posttranslational modifications of the ApoA1 residues on HDL [2].

HDL is built largely on a backbone of ApoA1, and given that it plays a vital role in reverse cholesterol transport (RCT), the transfer of excess cholesterol from peripheral tissues to the liver for excretion, it is atheroprotective. MPO can bind and oxidize HDL, alter its ability to accept cholesterol and negatively influence its anti-inflammatory properties [2, 3].

Psoriasis is a chronic inflammatory skin condition associated with immune activation, lipid dysfunction, and atherosclerosis. We previously reported elevated expression of MPO protein and a reduction in HDL function in a psoriasis cohort [4, 5].

However, a link between MPO levels and cholesterol efflux in human psoriasis has not been previously investigated. Therefore, the aim of this study was to evaluate the relationship between MPO concentration and RCT in a cohort of chronically inflamed psoriasis patients.

## Methods

The cohort of participants was selected using patients from the Psoriasis Atherosclerosis Cardiometabolic Disease Initiative (PACI: NCT01778569), January 2013 through November 2022. All participants provided written, informed consent, and the study protocols were approved by the institutional review board at the National Institutes of Health (NIH) following the Declaration of Helsinki. All patients underwent fasting blood draws for the assessment of lipid panels and hsCRP, which were performed in the NIH clinical center.

The capacity of patient HDL to perform reverse cholesterol transport was assessed in all patients by Cholesterol Efflux Assay (CEC) in J774 cells using a method previously described [5]. Plasma MPO levels were measured in a subset of 40 consecutive biologically naïve male patients at baseline using an in vitro enzyme-linked immunosorbent assay (cat # ab119605) following the manufacturer’s instructions (Abcam, Cambridge, MA). Samples for all assays were performed in duplicate.

### Statistical analysis

For this study, values are reported as the mean (standard deviation) for parametric values, median (interquartile range) for nonparametric values, and n (%) for categorical values. To elucidate the relationship between MPO and CEC, multivariate linear regression analysis was conducted including possible confounders such as absolute neutrophil count. CEC was log-transformed to meet the assumption for normality. Analyses were performed by StataIC 16 (Stata Corp., College Station, TX, USA) and R Statistical Software (version 4.1.3; R Foundation for Statistical Computing, Vienna, Austria). Two-tailed p values ≤ 0.05 were deemed statistically significant (bolded values).

## Results

Forty male psoriasis patients who were biologically naïve and not undergoing statin treatment were included in the study. Patients with MPO measurements were dichotomized by median MPO concentration (145 ng/mL). The clinical characteristics of each group are provided in Table 1. As expected, the subgroup with the highest MPO concentration also exhibited the highest absolute neutrophil count (high MPO 4.28 ± 1.04 vs. low MPO 3.30 ± 1.16; p = 0.01) but not monocytes (0.51 ± 0.13 vs. 0.50 ± 0.13; p = 0.11) or platelets (Table 1). Patients with high MPO had more systemic inflammation, as measured by hsCRP (high MPO 7.95 mg/L ± 11.70 vs. low MPO 1.8 ± 1.8; p = 0.08) and GlycA (440.36 µmol/L ± 94.52 vs. 384.8 ± 48.8). Characteristics such as psoriasis severity (high MPO 6.70 (5.05–14.05) vs. low MPO 6.90 (2.40-11.45) and BMI (26.05 (24.30–30.30) vs. 25.55 (24.30–28.40) were not significantly different.

**Table 1:** Psoriasis cohort stratified by median MPO concentration

Parameter	Low MPO [11.7–145)	High MPO (145.2–347]	P value
Demographic characteristics	N = 20	N = 20	
Age, y	50.90 ± 12.60	43.60 ± 12.10	0.07
Men, no. (%)	20 (100)	20 (100)	–
Framingham risk score	4.03 (1.24–5.58)	1.56 (0.25–7.76)	0.26
Race	15 (75)	14 (70)	> 0.99
Current smoker, no.	20 (100)	19 (95)	–
Hypertension, no.	18 (90)	17 (85)	0.66
BMI	25.55 (24.30–28.35)	26.05 (24.25–30.30)	0.55
Waist:hip ratio	0.99 (0.93–1.02)	0.96 (0.91–1.02)	–
Statin treatment, n (%)	0 (0)	0 (0)	–
PASI score	6.85 (2.40–11.45)	6.70 (5.05–14.05)	0.22
hsCRP, mg/L	1.80 ± 1.80	7.95 ± 11.70	0.08
GlycA, µmol/L	384.80 ± 48.80	440.36 ± 94.52	0.07
Neutrophils, K/µL	3.30 ± 1.16	4.28 ± 1.04	**0.01**
Monocytes, K/µL	0.50 ± 0.13	0.51 ± 0.13	0.11
Lymphocytes, K/µL	1.70 ± 0.55	1.73 ± 0.41	0.82
Neutrophil-lymphocyte ratio	2.0 ± 0.90	2.60 ± 0.90	0.09
Total cholesterol, mg/dL	187 ± 23.90	178.30 ± 39.50	0.41
LDL cholesterol, mg/dL	116.70 ± 27.16	111.30 ± 38.48	0.61
LDL particle number	1264.40 ± 299.54	1182.45 ± 416.35	0.48
LDL particle size	21.01 ± 0.63	20.93 ± 0.58	0.68
HDL cholesterol, mg/dL	52.6 ± 14.8	50.40 ± 10.80	0.60
HDL particle number	33.67 ± 5.80	31.68 ± 5.29	0.27
HDL particle size	9.02 ± 0.49	9.18 ± 0.49	0.25
HDL cholesterol efflux capacity	1.02 ± 0.20	0.89 ± 0.23	**0.05**
MPO concentration, ng/mL	58.80 (19.0–91.10)	187.8 (156.80–253.70)	**< 0.001**

**Fig. 1:**
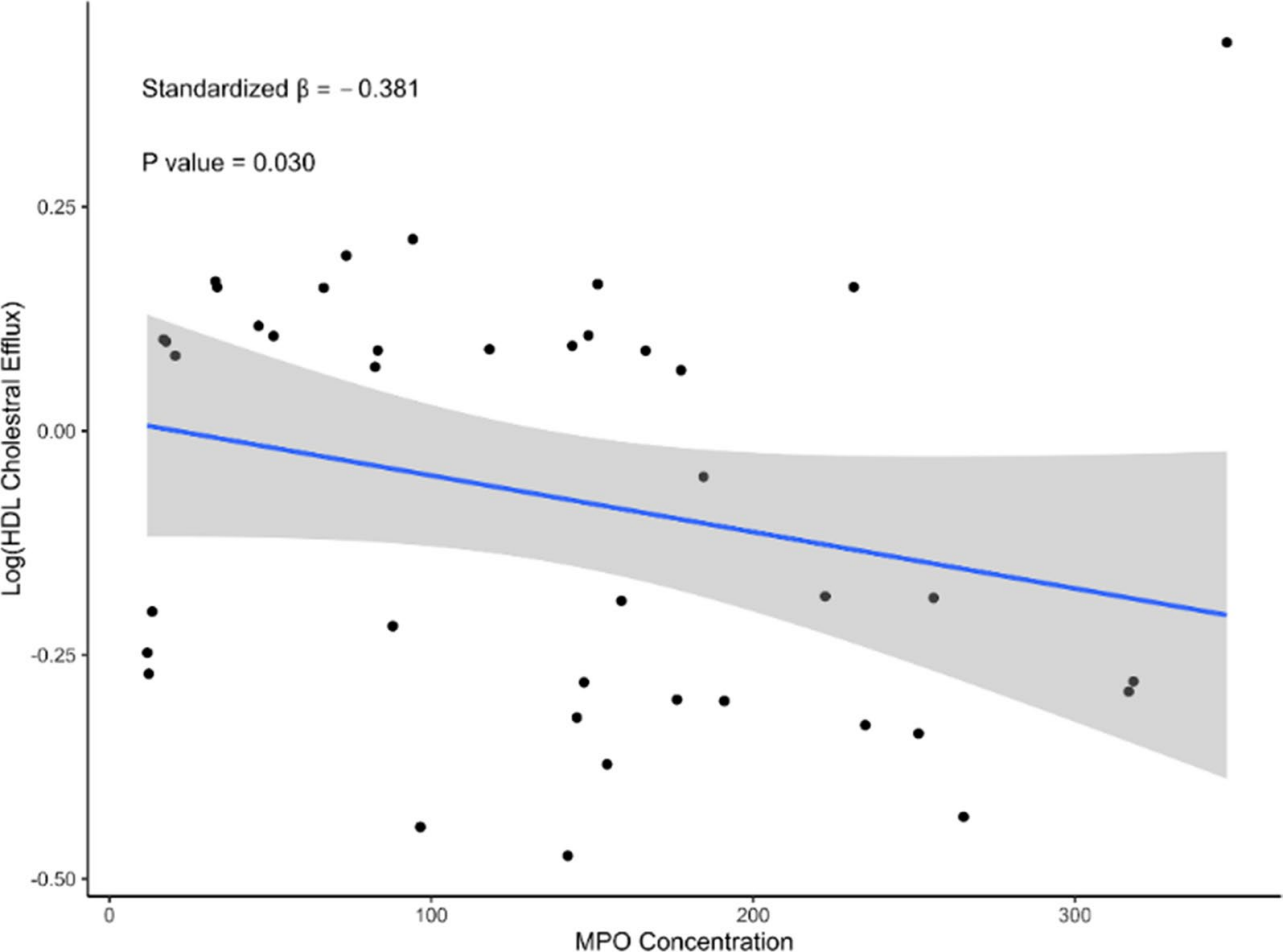
Relationship between MPO and cholesterol efflux capacity in the psoriasis cohort

Similarly, between the two groups, we found no significant difference in HDL or LDL cholesterol, particle number and size, or triglyceride concentration (Table 1). However, psoriasis subjects with high MPO levels had significantly lower CEC values than subjects with low MPO levels (high MPO 0.89 ± 0.23 vs. low MPO 1.02 ± 0.20; p = 0.05). Furthermore, using linear regression analysis on the whole cohort, we found that MPO concentration was negatively associated with CEC in models adjusted for age, sex, and absolute neutrophil count (β= − 0.381; p = 0.03) (Fig. 1).

## Discussion

In two separate studies, we previously showed that psoriasis patients have both elevated MPO concentrations and reduced CECs [4, 5]. Here, we show for the first time in a human population that these two observations are inversely associated; a high MPO concentration may suggest low CEC. The data concur with evidence that MPO can directly bind and oxidize HDL, leading to a dysfunctional HDL molecule [2, 3]. A high CEC indicates a high functioning HDL molecule considered to be atheroprotective. Hence, mechanisms to inhibit MPO expression or sequester it from HDL binding may be worthy of investigation as a therapeutic agent against early atherogenesis.

## Data Availability

Data used in this study are available from the corresponding author upon request.
